# The effect of a mobile application-based music intervention on pain levels and analgesic consumption following surgery: a randomized controlled trial

**DOI:** 10.1590/1806-9282.20251930

**Published:** 2026-06-29

**Authors:** Açelya Türkmen, Yasemin Özhanlı, İkbal Çavdar, Sevilay Erden, İlknur Tura, İsmail Furkan Başıbüyük, Mustafa Canikoğlu

**Affiliations:** 1 Çukurova University, Faculty of Health Sciences, Department of Surgical Diseases Nursing – Adana, Türkiye.; 2 Kocaeli University, Faculty of Health Sciences, Department of Surgical Nursing – Kocaeli, Türkiye.; 3 Istanbul Atlas University, Faculty of Health Sciences. Department of Nursing – Istanbul, Türkiye.; 4 Cukurova University, Balcalı Hospital Health Practice and Research Center – Adana, Türkiye.; 5 Kocaeli University, Faculty of Medicine, Department of Surgical Medical Sciences – Kocaeli, Türkiye.

**Keywords:** Orthopedic procedures, Pain, Nursing

## Abstract

**OBJECTIVE:**

The aim of this study was to evaluate the effect of mobile application-based musical tonalities delivered at different times of day on postoperative pain and analgesic consumption after orthopedic trauma surgery.

**METHODS:**

This randomized controlled study included 80 patients undergoing orthopedic trauma surgery at a university hospital. Tonalities (Rast, Nihavend, Neva) were delivered via an Android-based mobile application in the morning (T1), afternoon (T2), and evening (T3). Pain was assessed using the Numerical Rating Scale, and analgesic consumption was recorded.

**RESULTS:**

Baseline characteristics were largely comparable; however, body mass index (p=0.002) and marital status (p<0.001) differed between groups. Pain scores decreased significantly over time in both groups (p<0.001). After Bonferroni correction, baseline pain did not differ between groups, whereas post-intervention pain scores differed at all post-intervention time points (T1b, T2b, T3b; p<0.0083), with lower scores in the control group. Analgesic consumption did not differ significantly between groups (p>0.05). In robust regression analyses controlling for baseline Numerical Rating Scale, body mass index, and marital status, the intervention group showed significantly higher Numerical Rating Scale scores at all measurement times, and baseline Numerical Rating Scale was the strongest predictor of postoperative pain.

**CONCLUSION:**

Postoperative pain decreased over time in both groups, and baseline pain level was the strongest determinant of postoperative pain perception; no significant between-group difference was observed in analgesic consumption.

## BACKGROUND

Postoperative pain is common after trauma surgery, with acute pain reported in up to 80% of patients and moderate to severe pain in approximately 70%^1^. Inadequately controlled pain may lead to hypoventilation, increased oxygen demand, sleep disturbance, anxiety, and delayed recovery^2^. Trauma surgery is associated with severe pain due to extensive injury to nerves, muscles, bones, and connective tissues^3,4^. Although pharmacological approaches such as opioids, non-opioids, and nerve blocks are routinely used, opioid-related adverse effects and addiction risk highlight the need for complementary non-pharmacological strategies^3,4^.

Music is a non-invasive, low-cost, and safe intervention shown to reduce postoperative pain^5,6^ and analgesic consumption^7^. Clinical guidelines recommend incorporating music-based cognitive–behavioral interventions into multimodal pain management^6,7,8^. Music alleviates both physiological and psychological aspects of pain by promoting relaxation, enhancing coping, and reducing analgesic-related side effects^9^. Analgesic effects of music are mediated through distraction, modulation of physiological arousal, hormonal responses, and emotional regulation and may vary according to rhythm, tempo, tonality, and individual familiarity or cultural background^9,10^. Calm, slow-tempo music is generally more effective in pain reduction than fast or intense music^10,11^.

Most previous studies have selected music based on patient or researcher preference^6,12,13,14^. However, to the best of our knowledge, no music intervention in the literature has been applied according to patients’ emotional states or at different times of the day following trauma surgery.

## METHODS

**Figure 1:**
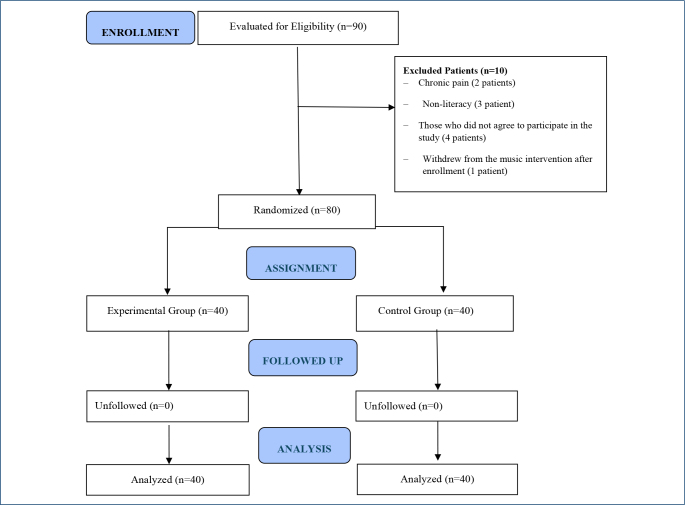
Consolidated Standards of Reporting Trials 2010 flow diagram.

Study design and participants: This randomized controlled study was conducted with patients who underwent orthopedic trauma surgery at a university hospital. The sample consisted of 80 patients who provided informed consent and met the inclusion criteria. Eligible participants were aged ≥18 years, had no mental or auditory impairments or chronic pain, had undergone orthopedic trauma surgery, and were able to read and write. Patients who declined participation (n=4), had chronic pain (n=2), were illiterate (n=3), or withdrew from the music intervention after enrollment (n=1) were excluded (Figure 1).

The study’s power was calculated using the G*Power 3.1.9 program for post-hoc power analysis with a 5% error rate and a sample size of 80 (40=Control, 40=Experimental). This analysis showed that the study achieved 90% power for Measure 1 (effect size=0.738) and 97% power for Measures 2 and 3 (effect sizes=0.879 and 0.888).

Instruments: Data were collected using an android-based mobile music application developed by a software developer. The application included tabs for patient information, pain assessment, analgesic consumption, and music intervention.

Android-based mobile music application: The application was used to assess the effects of music intervention on pain and analgesic consumption in orthopedic trauma surgery patients and included modules for patient data entry, the Numerical Rating Scale (NRS), and music delivery.

Information Form: Developed by the researchers based on the literature^6,9,15^, this form collected demographic, surgical, and analgesic-related data.

NRS: Pain intensity was measured using the NRS, ranging from 0 (no pain) to 10 (unbearable pain).

**Figure 2:**
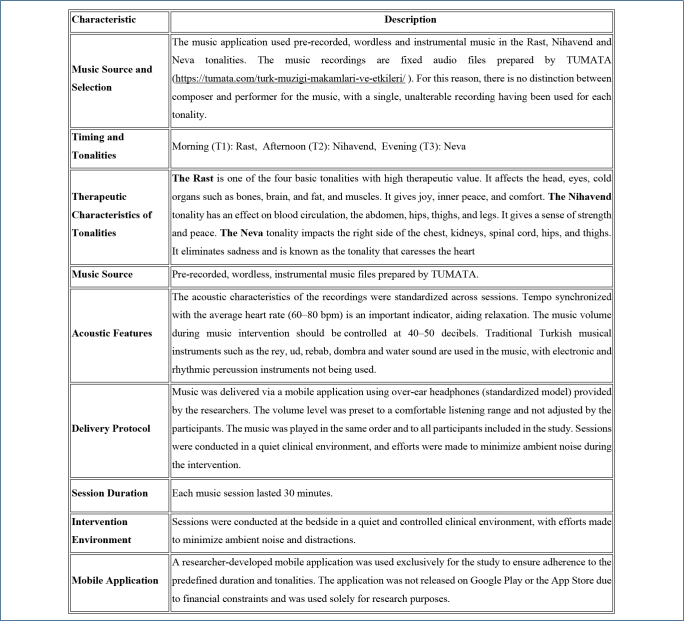
Music intervention protocol.

Music intervention: Musical tonalities were selected in consultation with TUMATA^16^ (Turkish Music Research and Promotion Group). Rast was applied in the morning (T1), Nihavend in the afternoon (T2), and Neva in the evening (T3). Tonality characteristics are detailed in the intervention protocol (Figure 2).

Ethics statement: Ethical approval was obtained from the institutional ethics committee (No: 24.12.2020-241). Written informed consent was obtained from all participants in accordance with the Declaration of Helsinki. The study was registered at ClinicalTrials.gov (NCT05424211) and reported in compliance with the Consolidated Standards of Reporting Trials guidelines.

Implementation of the study: At baseline, patients were informed about the study and provided written and verbal consent. Data were collected on the first postoperative day using an Android-based mobile application, through which participants were assigned to the experimental or control group.

Experimental group: The descriptive characteristics of patients in the experimental group were recorded on the application. The experimental group received a standardized Turkish music tonalities-based music application via a mobile application. The music intervention was implemented according to the protocol (Figure 2) using headphones via a mobile application that was developed for research purposes. The music was played in the same order and to all participants included in the study. Patients listened to the tonality uploaded to their mobile phones using the Android-based mobile application three times for 30 min in the morning, afternoon, and evening. Pain levels were assessed and recorded immediately before and 1 h after the music intervention. The total amount of analgesic consumption used was recorded at the end of the day. Mobilization status was assessed by recording the number of ambulation attempts during the postoperative period. The intervention was conducted at patients’ bedsides in the postoperative period in a quiet, controlled environment without visitors, and no other interventions or distracting stimuli were present during the music session.

Control group: Control-group patients’ information was also recorded on the application, but their routine analgesic drug administration was not interfered with. The control group’s pain scores were assessed at the same frequency and at the same times of day as the experimental group. Total amount of analgesic consumption used was recorded at the end of the day.

Randomization: Participants were randomized 1:1 using computer-generated block randomization by an independent statistician, with allocation concealed in sequentially numbered, opaque, sealed envelopes. Blinding of participants and practitioners was not feasible due to the behavioral intervention; however, the statistician performing the analyses was blinded to group allocation.

Statistical analysis: Statistical analyses were conducted using International Business Machines-Statistical Package for the Social Sciences Statistics Premium Grad Pack Student V.30.0 and RStudio (V.2024.12.1). Descriptive statistics were presented as frequencies, percentages, means, standard deviations, medians, and interquartile ranges (IQRs). Data normality was assessed using histograms, Q–Q plots, and the Shapiro-Wilk test. As pain scores did not meet normality assumptions, non-parametric tests were applied: the Mann-Whitney U test for between-group comparisons, the Friedman test for with-in-group changes over time, and the Wilcoxon Signed-Rank test for pre–post comparisons within intervention sessions. Bonferroni correction was used to control for Type I error, with an adjusted significance level of p<0.0083 (0.05/6). Additionally, robust regression analysis (Method of Moments [MM]-estimator, lmrob) was performed to adjust for baseline confounding variables and to estimate the independent effect of the intervention. Robust regression analysis was applied to enhance the reliability of the model (RStudio—robustbase library; MM-estimator, lmrob). A significance level of p<0.05 was adopted unless otherwise specified.

## RESULTS

Baseline homogeneity analyses showed no significant between-group differences in age, mobilization frequency, gender, or educational status (p>0.05). However, body mass index (BMI) (p=0.002) and marital status (p<0.001) differed significantly between groups (Table 1).

**Table 1:** Sample characteristics, overall and by froup (n=80).

Demographic variables	Music group		Control group		Statistic
	(n=40)		(n=40)		p
	x̄± SD/n	M (IQR)/%	x± SD/n	M (IQR)/%	
Age (year)	56.37±17.58	61.0 (24.0)	48.35±21.17	43.50 (39.50)	0.102^*^
Intervention (min.=18; maks.=83), control (min.=21; maks.=90)					
BMI (kg/m^2^)	30.79±6.24	30.08 (6.40)	27.02±3.79	27.16 (4.55)	0.002^*^
Mobilizations					
Morning (T1)	0.43±0.59	0.0 (1.0)	0.56±0.90	0.0 (1.0)	0.848^*^
Noon (T2)	1.00±1.06	1.0 (1.0)	1.28±1.28	1.0 (2.5)	0.458^*^
Evening (T3)	1.58±1.32	1.0 (1.0)	2.65±2.58	2.0 (3.5)	0.162^*^
	**n (%)**		**n (%)**		
Gender					
Female	21 (52.5)		14 (35.0)		
Male	19 (47.5)		26 (65.0)		0.176^***^
Marital status					
Married	31 (77.5)		16 (40.0)		
Single	9 (22.5)		24 (60.0)		<0.001^***^
Education					
Illiterate	3 (7.5)		8 (20.0)		
Literate (no formal schooling)	3 (7.5)		5 (12.5)		0.068^**^
Primary education	22 (55.0)		10 (25.0)		
High school	6 (15.0)		11 (27.5)		
University	6 (15.0)		6 (15.0)		

x̄: mean; SD: standard deviation; n: number of observations; %: percentage; Min: minimum value; Max: maximum value; M (IQR): median (interquartile range, Q3–Q1).

^*^Mann-Whitney U test standardized Z-table value

^**^Pearson chi-square test value,

^***^Continuity correction chi-square test value. BMI: body mass index.

Pain scores changed significantly over time (T1a–T3b) in both groups [experimental group: χ^2^(F)=43.412, p<0.001; control group: χ^2^(F)=90.843, p<0.001]. Related samples Wilcoxon Signed Rank tests indicated that post-intervention pain scores decreased significantly compared with pre-intervention levels in both groups (Table 2).

**Table 2:** Between-group comparison of Numerical Rating Scale pain levels before and after the intervention, and analgesic consumption (n=80).

Interventions	Music group (n=40)			Control group (n=40)			Statistics	
	x± SD		M (IQR)	x± SD		M (IQR)	Z^a^	p
Intervention 1								
Before (T1a)	5.20±1.47		5.00 (2.00)	6.05±2.43		6.00 (3.00)	2.553	0.011
After (T1b)	3.75±1.72		3.00 (1.50)	2.45±1.80		3.00 (3.00)	-2.953	0.003
Test; p	Z^b^=-4.163, p<0.001			Z^b^=-5.328, p<0.001				
Intervention 2								
Before (T2a)	5.33±2.19		6.00 (3.00)	4.50±2.63		6.00 (3.00)	-1.325	0.185
After (T2b)	3.85±2.44		3.50 (3.00)	1.95±1.84		2.00 (3.00)	-3.634	<0.001
Test; p	Z^b^=-3.894, p<0.001			Z^b^=-4.823, p<0.001				
Intervention 3								
Before (T3a)	4.85±1.78		5.00 (2.00)	3.53±2.68		4.00 (5.00)	-2.301	0.021
After (T3b)	3.48±2.18		3.00 (3.00)	1.70±1.81		2.00 (2.00)	-3.735	<0.001
Test; p	Z^b^=-4.545, p<0.001			Z^b^=-4.407, p<0.001				
Test; p	ꭓ^2^_(F)_=43,412, p<0.001			ꭓ^2^_(F)_=90.843, p<0.001				
**Analgesic consumption among groups**								
**Analgesics**	**n**	**x**±**SD**	**M (IQR)**	**n**	**x**±**SD**	**M (IQR)**		**p**
Paracetamol (mg)	28	1,645.54±618.77	2,000.0 (1,000)	32	1,426.56±517.44	1,000.0 (1,000)		0.142^*^
Diclofenac Sodium (mg)	20	155.00±222.37	75.0 (75)	17	88.24±29.47	75.0 (0)		0.311^*^
Tramadol hydrochloride (mg)	4	118.75±55.43	100.0 (63)	7	128.57±48.80	100.0 (100)		0.648^*^
Ibuprofen (mg)	4	237.50±187.64	237.5 (325)	8	450.00±141.42	400.0 (0)		0.154^*^

n: number of observations; x̄: mean; SD: standard deviation; M (IQR): median (interquartile range, Q3–Q1); NRS: Numeric Pain Scale (0–10); T1a, T1b, T2a, T2b, T3a and T3b: music intervention points. Z^2^(F)=Friedman test;

Z^a *^Mann-Whitney U-test standardized Z-table value; Z^b^: related samples Wilcoxon signed-rank test; mg: milligram.

Although baseline pain was higher in the control group than in the experimental group at T1a, this difference was not statistically significant after Bonferroni correction (p<0.0083); likewise, the difference at T3a did not remain significant after correction. In contrast, between-group differences were significant at all post-intervention time points (T1b, T2b, and T3b) (p<0.0083), with lower pain scores in the control group (Table 2).

Total analgesic consumption did not differ significantly between groups (p>0.05) (Table 2).

In robust regression analyses controlling for pre-test NRS score, BMI, and marital status, the experimental group had significantly higher final NRS pain scores than the control group at all measurement times. Pre-test NRS was a strong predictor across models (T1: B=0.48; T2: B=0.57; T3: B=0.63; p<0.001), whereas BMI was significant only at T2 and marital status only at T3. The pre-test NRS score model explains 16% of the variance (Table 3).

**Table 3:** Results of the robust regression analysis of final Numerical Rating Scale pain scores.

Variables	T1 B (95%CI)	t	p	T2 B (95%CI)	t	p	T3 B (95%CI)	t	p
Group (intervention)	1.66 (0.99–2.33)	4.91	<0.001	0.91 (0.04–1.78)	2.08	0.041	1.26 (0.47–2.04)	3.18	0.002
Pre-NRS	0.48 (0.33–0.64)	6.12	<0.001	0.57 (0.44–0.71)	8.28	<0.001	0.63 (0.47–0.79)	7.82	<0.001
BMI	-0.01 (-0.09–0.06)	-0.36	0.717	0.11 (0.02–0.21)	2.31	0.024	-0.01 (-0.08–0.06)	-0.25	0.802
Marital status (marriage)	-0.18 (-0.86–0.50)	-0.53	0.599	-0.08 (-0.88–0.73)	-0.19	0.849	-0.82 (-1.57–0.08)	-2.20	0.031

B: regression coefficient. CI: confidence intervals. BMI: body mass index; NRS: Numerical Rating Scale.

## DISCUSSION

This randomized controlled trial evaluated the effect of a mobile application–based music intervention on postoperative pain and analgesic consumption. Although robust regression analyses showed higher NRS pain scores in the intervention group at all time points, adjustment for baseline pain levels and confounding variables revealed that the reduction in pain over time was greater in the intervention group compared with the control group.

Previous studies indicate that effective pain-relieving music should be harmonious, repetitive, instrumental, and easy to listen to^6,12,13,14^. Findings from TUMATA^16^ indicate that musical tonalities are effective in reducing musculoskeletal pain, and variations in acoustic features such as tempo, mode, and loudness influence patients’ emotional responses^17^. In Schneider’s study, patients selected their own music, which mainly consisted of culturally familiar, melancholic pieces with slow tempos of approximately 60–80 beats. There is no consensus on music selection in postoperative pain management; therefore, a wide range of music genres has been used in the literature^6,12,13,14,18^. The literature suggests that culturally familiar, self-selected music enhances relaxation, and patient involvement in perioperative care and decision-making improves patient outcomes^19^. In this context, our study is valuable in terms of tonalities used in the music intervention, individuals’ feeling of safety and relaxation, and monitoring the changes in pain level thanks to muscle relaxation.

The literature indicates that music interventions significantly reduce postoperative pain^6,9,20^. A study was conducted with orthopedic patients, which found that music therapy significantly reduced pain scores^21^. However, Akelma et al.^22^ found that music intervention did not yield a statistically significant difference between experimental and control groups. In our study, it was determined that pain scores in both groups showed a significant change over time (from T1a to T3b). Post-intervention pain levels in both groups decreased to a statistically significant degree compared to pre-intervention levels. However, the pain trajectories differed between groups, with lower pain scores observed in the control group. These differences may be explained by baseline confounders, particularly higher BMI and lower mobilization frequency in the experimental group, which may have contributed to a slower reduction in pain perception.

There were some meta-analysis studies and systematic reviews in the literature that supported the use of music to help reduce perioperative pain and analgesic consumption in patients undergoing orthopedic surgery^1,9,12,21^. In some studies reported that music intervention did not affect the analgesic dose used post-operatively^20^. In our study, it was observed that no statistically significant differences were found between the groups in terms of analgesic consumption (p>0.05). However, lower opioid and nonsteroidal anti-ınflammatory drug consumption observed in the music group suggests that music intervention may have a complementary effect alongside standard analgesic therapy. The high pain profile of orthopedic surgeries and the effective implementation of postoperative analgesic protocols may have limited the impact of music on analgesic consumption; therefore, music should be considered a complementary care approach that supports, rather than replaces, pharmacological pain management.

Robust regression analyses showed that, after controlling for baseline NRS score, BMI, and marital status, final NRS pain scores in the experimental group remained significantly higher than those of the control group at all time points. This finding suggests that baseline pain intensity is a strong determinant of subsequent pain perception and may partially mask the observable effect of nonpharmacological interventions. These results suggest that baseline clinical characteristics should be taken into account when interpreting the effects of complementary interventions such as music therapy.

## CONCLUSION

In conclusion, postoperative pain decreased over time in both groups. However, after adjusting for baseline pain, BMI, and marital status, the music intervention was found to have no significant effect on pain or analgesic consumption. Baseline pain was found to be the strongest predictor of postoperative pain. Interpretation of these findings is limited by the short duration of the music intervention and baseline differences between groups despite randomization. Nevertheless, the use of music in the early postoperative period may be considered a supportive, complementary approach in orthopedic patients. Future studies should evaluate music interventions applied throughout the perioperative period.

## Data Availability

The datasets generated and/or analyzed during the current study are available from the corresponding author upon reasonable request.
